# Cohesive or Mechanical Force

**Published:** 1881-05

**Authors:** T. D. Shumway


### ARTICLE VI.

### *Cohesive or Mechanical Force.*

BY T. D. SHUMWAY, PLYMOUTH, MASS.

In the use of the mallet in gold filling it is assumed that the cohesive property unaided is not sufficient to give that strength which is necessary to resist the force con stantly brought to bear against it. It is also assumed that mechanical force is indispensable to consider or impact the gold, so that the filling will not be porous, and thus absorb moisture, and induce subsequent decay. We wish to show that this assumption, on which the whole theory of mallet practice is based, is contrary to well-recognized principles, that a more perfect union is not possible by any such process, and that the every day experience of every dentist with any other material than gold is witness to this truth.

If we had a lump of rock salt to dissolve in the shortest possible time, we would first reduce it to powder, that the water might act the more readily upon all its surfaces. It was the attraction of cohesion that held the grains of salt in one body, and it is mechanical force applied to that body which aids disintegration. The teeth are among the great disintegrators. Attention is the application of mechanical force to destroy cohesion. How absurd to assume that the cohesive property in wheat is to be improved by putting it under the millstone, or when it has been taken as food and assimilated, circulation may be increased by pounding the heart with a sledge hammer; or again, to expect to hasten the growth and development of the tree by forcing the sap with a force pump.

But these illustrations are no more absurd than the assumption that a more perfect union of the particles of cohesive gold is obtained by the application of mechanical force. Throughout all nature we observe a tendency in matter to run into symmetric forms. All homogeneous particles aggregate to make the unit. We all recognize this law, and know that the application of extraneous force is opposed to the union, yet when we use cohesive gold, in which this tendency to unite is developed in the highest degree, we reverse the order of nature and deny the existence of the law. The wonder is, not that gold has developed into a dangerous filling material, but that under such treatment the teeth have not entirely broken down.

This shows the strong recuperative tendency in the teeth, and proves that a rational or more scientific method would have wrought the most beneficial results. But, it is urged, the gold is obstinate and refuses to yield to persuasion; that force becomes a necessity to bring the particles together to perfect the union. This, it is claimed, is the practical result, while the other is only a fine-spun theory. Now, this obstinacy in the gold is the best reason why it should not be subjected to the malleting process. If it does not yield willingly, it should not be made to yield by force. If cohesion is not the result of persuasion, there can be no natural union. When force is applied, a mechanical union takes the place of natural cohesion, for both cannot exist in the same body at the same instant.

All bodies are made up of minute particles, and these particles, as already stated, tend towards a particular form. These forms constitute the physical properties of matter. But there is another force as that of cohesion, acting under certain conditions, upon all the atoms of which these bodies are composed. All matter is endowed with an affinity or chemical attraction, exerted between its minutest particles, which, when combined, form new bodies with different properties. While cohesion is always opposed to the action of affinity, chemical attraction, like cohesion, acts at an insensible distance. Contact is necessary to effect combination, and mechanical action always favors affinity. Now, when cohesive gold refuses to yield to persuasion, and force is employed to bring the particles together, mechanical union takes the place of cohesion, and combination of different elements must be the inevitable result. The same rule does not apply to gold in the non-cohesive form, for the reason that chemical attraction is exerted with different degrees of force, sometimes with great energy, at others feebly or not at all. This is why non-cohesive gold preserves the tooth, when cohesive gold does not. The frequent inquiry as to the cause of the discoloration of so many cohesive gold fillings, and the various reasons given therefor, show a want of appreciation of this subtle but very important principle. Many dentists are continually changing from one manufacture of gold foil to another, in the hope of finding an article of greater purity that will not discolor. Now, the fault is not with the gold, but with the method of working it. The gold may be of virgin purity, but if it is subjected to mechanical force when in the excited or cohesive state, it will develop entirely different properties by chemical attraction.

Every dentist has the means at hand to determine the purity of gold foil. Until he has informed himself of the methods of testing the quality of gold, he should not attempt to make use of the cohesive principle to arrest decay in teeth with any expectation of satisfactory results. Gold in the cohesive state is in the highest degree susceptible, and eager to combine with any material for which it has an affinity. There is no other metal that combines more readily with gold than iron. When once united it is separated with difficulty. The combining of iron with gold changes its properties. This combination makes the gold stiff, hard and unyielding. The more force applied under these conditions, the greater will be the change. It is this change in the nature of gold in the cohesive state, by the use of iron or steel instruments with mechanical force, combined with a want of perfect adaptation by reason of a change of properties that produces discoloration in the filling.

To charge the blame to the gold-beater, for a result which is the effect of a disregard of a natural law, simply displays a lack of knowledge on the part of the dentist. The action of affinity, by changing the properties in cohesive gold, facilitates the disintegration of the surrounding tooth structure. This is what gave rise to the incompatibility or electro-chemical theory. To deny the conclusions of Drs. Chase and Palmer, after careful investigation, that cohesive gold used with mechanical force is not compatible with dentos, is to maintain that the order of nature can be reversed with impunity. A method of practice so contrary to natural law must result in compatibility and consequent failure. The way in which the "new departure" has been attacked by those devoted to the use of the mallet, gives evidence of a want of system based upon knowledge and demonstration. The inconsistencies of the advocates of a theory should have no weight in scientific investigation after truth.

In closing we wish to remark upon the statement that the theory of practice we have endeavored to define, involves too many fine-spun points to be of practical value. The operations the dentist is called upon to perform are confined to organs as sensitive as any in the whole human economy. In the performance of them the patient has a right to demand that delicacy shall be combined with efficiency. It is no reason because all the force that could be exerted has been employed, that it must always continue. Machinery may be used to advantage in operations upon the teeth, but the use of it should be seasoned with moderation and judgment. In the calling of a dentist the operator should be schooled and disciplined in delicacy of touch. No theory should be thought too fine spun, and no method too delicate that promises to mitigate suffering and stay the progress of decay.—*Dental Office and Laboratory.*

---

